# Unconsciously Perceived Fear in Peripheral Vision Alerts the Limbic System: A MEG Study

**DOI:** 10.1371/journal.pone.0008207

**Published:** 2009-12-09

**Authors:** Dimitri J. Bayle, Marie-Anne Henaff, Pierre Krolak-Salmon

**Affiliations:** 1 INSERM, U821, Lyon, France; 2 Université Lyon 1, Lyon, France; 3 Diagnostic Imaging, Research Institute, Hospital for Sick Children, Toronto, Ontario, Canada; 4 Hospices Civils de Lyon, Lyon, France; University of Minnesota, United States of America

## Abstract

**Background:**

In ecological situations, threatening stimuli often come out from the peripheral vision. Such aggressive messages must trigger rapid attention to the periphery to allow a fast and adapted motor reaction. Several clues converge to hypothesize that peripheral danger presentation can trigger off a fast arousal network potentially independent of the consciousness spot.

**Methodology/Principal Findings:**

In the present MEG study, spatio-temporal dynamics of the neural processing of danger related stimuli were explored as a function of the stimuli position in the visual field. Fearful and neutral faces were briefly presented in the central or peripheral visual field, and were followed by target faces stimuli. An event-related beamformer source analysis model was applied in three time windows following the first face presentations: 80 to 130 ms, 140 to 190 ms, and 210 to 260 ms. The frontal lobe and the right internal temporal lobe part, including the amygdala, reacted as soon as 80 ms of latency to fear occurring in the peripheral vision. For central presentation, fearful faces evoked the classical neuronal activity along the occipito-temporal visual pathway between 140 and 190 ms.

**Conclusions:**

Thus, the high spatio-temporal resolution of MEG allowed disclosing a fast response of a network involving medial temporal and frontal structures in the processing of fear related stimuli occurring unconsciously in the peripheral visual field. Whereas centrally presented stimuli are precisely processed by the ventral occipito-temporal cortex, the related-to-danger stimuli appearing in the peripheral visual field are more efficient to produce a fast automatic alert response possibly conveyed by subcortical structures.

## Introduction

There is no question that human behaviour is affected by environmental input. What is less known is that most environmental stimuli are not consciously perceived [1], [2], yet they nevertheless modulate behaviour [2]–[4]. The processing of unconsciously perceived stimuli is particularly important for visually salient or arousing stimuli. Thus, facial emotional expressions, particularly fear, can be processed in the absence of awareness [5], [6], triggering changes in skin conductance [7], [8] and judgment of subsequent targets [9], [10]. As fear is linked to danger, detecting fear in the environment, even unconsciously, enhances vigilance and alertness, which is essential to produce fast and adapted behavioural reactions.

Facial expression detection is mediated by distributed neural systems [11] including many of the brain structures involved in processing visual stimuli in general. The main pathway involves the lateral geniculate nucleus, the striate cortex, and parietal and temporal extrastriate cortices. Elementary visual feature processing related to face detection induces occipital activity around 90 ms [12]. Following temporal cortex reaction is mainly disclosed by fusiform gyrus related to structural face processing occurring around 170 ms [13]–[15] and superior temporal gyrus related to changeable facial feature analysis, particularly to facial expressions [16]. More anterior structures like amygdala and orbito-frontal cortex are reported to be activated later [17].

In parallel to this main visual pathway, a second pathway has been suggested to process danger-related stimuli, in this case fearful faces [6], [18]. This pathway would involve a retino-tectal route and subcortical structures, mainly the superior colliculus, the pulvinar, and the amygdala [19]. This route may bypass the primary visual cortex and is thought to be limited to a relatively coarse and automatic processing, especially of visual transient and highly salient visual features [11]. The residual detection capacities of blindsight patients in their blind hemifield after a striate visual cortex lesion validate the existence of a subcortical route [20]. Interestingly, in a forced choice task, a blindsight patient was able to discriminate emotional faces in his blind hemifield without explicit knowledge. In this patient, fearful faces produced amygdala activation mediated by the superior colliculus and the pulvinar [21]. Another study in a cortically blind patient has shown a correct guessing for emotional faces and not for other emotional stimuli [22]. The patient's right amygdala was activated during the unconscious processing of emotional faces. Thus, behavioural and neuroimaging data suggest that non-consciously perceived facial expressions may access the amygdala and frontal cortex via a subcortical visual route bypassing the striate cortex [23].

Animal studies established that colliculus is largely fed by magnocellular cells [24], [25]. Consequently, the colliculo-thalamo-amygdalar pathway is particularly sensitive to the visual properties conveyed by the magnocellular system, i.e. low spatial frequencies and rapid, dynamic stimuli. Interestingly, recent studies have demonstrated that low spatial frequencies are particularly implicated in fearful faces perception [26], [27], suggesting an important role of the magnocellular system in threat-stimulus perception. The magnocellular system is essentially afferented by the peripheral retina [28]. Moreover, the colliculus and the pulvinar are oculomotor structures involved in saccade production towards targets in the peripheral visual field and are thus particularly tuned to peripheral stimulations. In ecological situations, danger often appears first in the peripheral visual field. A rapid reaction would influence survival and thus is likely driven by a fast and phylogenetically old system. By stimulating mainly the magnocellular system, peripheral fearful faces would trigger a fast brain response, possibly conveyed by the subcortical colliculo-thalamo-amygdalar route. Indeed this last route shows these properties of rapidity [18], automaticity [6], emotional detection capacity [27] and peripheral preference [29].

Subcortical structures are activated by fearful faces when their presentation is subliminal and central [6], [18], [30], [31], but the questions of how and how fast the brain processes emotional faces briefly presented in the peripheral visual field have not been addressed. By automatically recruiting specific brain regions in the first steps of visual analysis, peripheral threatening stimuli should allow a fast and adapted defensive reaction. We hypothesized in this study that fearful faces unconsciously perceived in the peripheral visual field, by stimulating mainly magnocellular cells, would particularly trigger a fast neuronal response implicating the colliculo-thalamo-amygdalar pathway and then, the frontal cortex. We used the Magnetoencephalography (MEG) which combines an excellent temporal and good spatial resolution [32] to record brain reaction to centrally versus peripherally very briefly presented fearful stimuli.

## Results

The 2 by 2 ANOVA (2 spatial positions, 2 facial expressions) performed on the 3D activation maps resulting from source activation analysis (see further description in the methods) revealed statistically significant interactions between the 2 factors in several brain regions, encompassing a large part of the four cerebral lobes. The interaction was highly significant in the right frontal lobe, the central occipital region and in both temporal lobes. The Interaction was also significant in the left frontal lobe and in the left inferior and right superior parietal regions. In all regions with a significant interaction between spatial expressions and spatial positions, the differences of activations between fearful and neutral faces were tested (one sampled t test) for the central and the peripheral presentation. Structures exhibiting a statistically differential response to stimuli between fearful and neutral faces are listed in Table 1 and Table 2, respectively for central and peripheral presentation condition.

**Table 1:** Brain areas more activated by fearful than by neutral centrally presented faces in the three analyzed time windows.

Time windows windows windows windows	Brain regions	side	Talairach coordinates				
			x	y	z	Student-t	Volume
**80 to 130 ms**	Inferior temporal sulcus	L	−50	−39	−6	4.82	2
	Inferior temporal gyrus	L	−40	−2	−38	3.57	4.5
**140 to 190 ms**	Post-central gyrus**	R	45	−17	47	5.46	11
	Middle temporal gyrus**	R	64	−30	11	5.41	8
	Superior temporal gyrus*	R	54	−62	26	4.89	10.5
	Inferior temporal gyrus	L	−59	−25	−16	4.47	4.25
	Fusiform gyrus	R	40	−74	−17	3.56	2.25
	Inferior occipital gyrus	L	−35	−83	−13	3.5	4.25
**210 to 260 ms**	Middle temporal gyrus**	R	50	−30	−11	5.91	3.5
	Inferior parietal lobule	L	−64	−37	30	4.53	1.25
	Middle frontal gyrus	L	−20	53	−7	3.5	1.5

For each activation cluster, the Talairach coordinates correspond to the voxel of maximal intensity obtained after the ERB analyses, the volumes are expressed in cm^3^. The threshold is set at uncorrected p<0.01 (*p<0.005, **p<.001).

**Table 2:** Brain areas more activated by fearful than by neutral peripherally presented faces in the three analyzed time windows.

Time windows	Brain regions	side	Talairach coordinates				
			x	y	z	Student-t	Volume
**80 to 130 ms**	Precuneus**	R	15	−62	26	5.23	5
	Post and pre-central gyrus*	R	54	−13	24	4.72	8.5
	Uncus and amygdala	R	31	−11	−33	4.43	3.75
	Medial frontal gyrus/anterior cingulate	R	1	54	11	4.19	2.75
**140 to 190 ms**	Medial frontal gyrus*	R	0	59	6	4.99	1.75
	Post and pre-central gyrus*	R	59	−9	14	4.6	8.5
	Inferior parietal lobule	L	−59	−37	34	4.02	1.25
	Middle occipital gyrus	L	−35	−92	0	3.78	4
**210 to 260 ms**	Post-central gyrus	L	−59	−18	−19	7.19	1.25
	Supramarginal gyrus	L	−59	−42	34	3.62	1.25

For each activation cluster, the Talairach coordinates correspond to the voxel of maximal intensity obtained after the ERB analyses, the volumes are expressed in cm^3^. The threshold is set at uncorrected p<0.01 (*p<0.005, **p<.001).

### Central presentation

Table 1 reports these structures across the three time windows, for central presentation. In the earliest time window (80 to 130 ms), significantly (p<0.01) higher responses to fear were found only in the left hemisphere, in the inferior temporal sulcus and the anterior part of the inferior temporal gyrus. Between 140 and 190 ms, the left inferior temporal region was still more active (p<0.01) for fearful faces as well as the right post-central gyrus and right temporal regions including the middle and superior temporal gyri, the right fusiform gyrus, and finally the left occipital gyrus. In the third time window (210–260 ms), a statistically significant difference of activation was maintained in the right middle temporal region and simultaneously appeared in the left inferior parietal and middle frontal regions.

### Peripheral presentation

**Figure 1:**
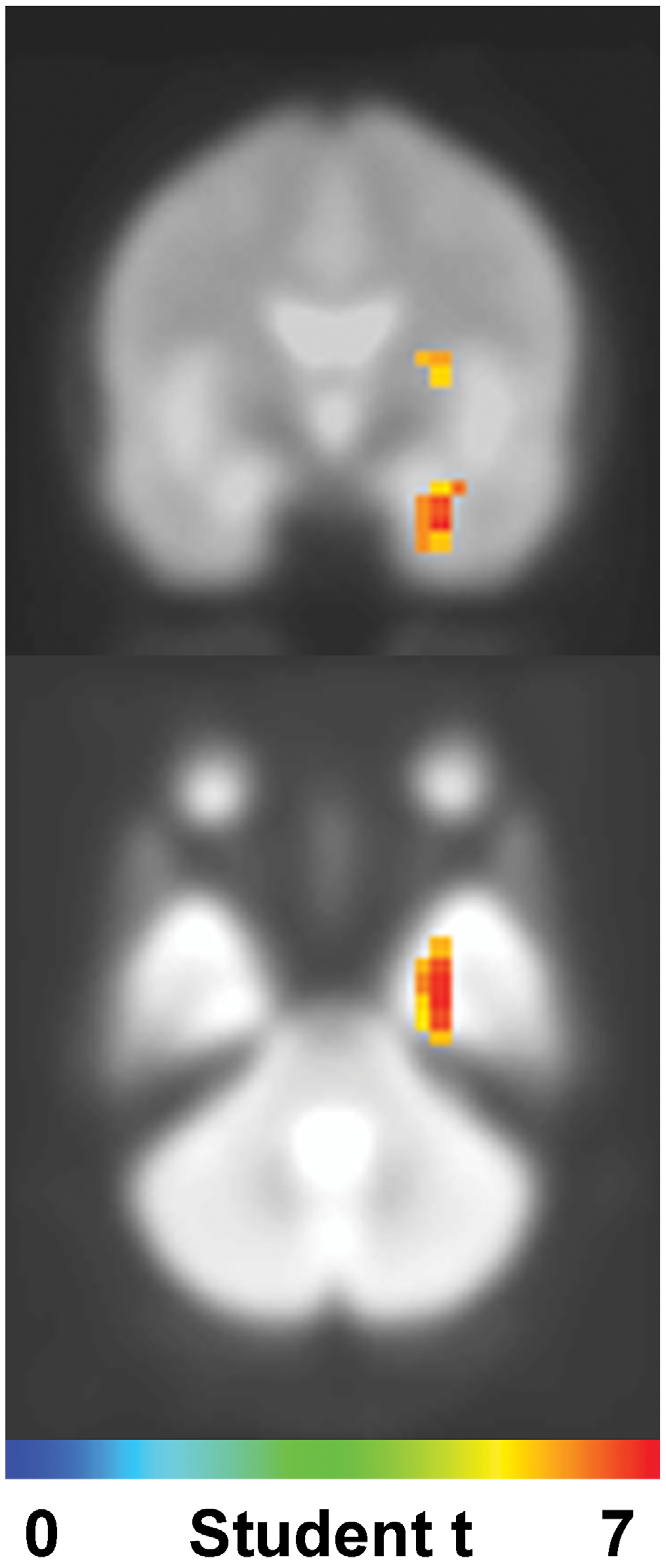
Group source analysis. The student-t statistic 3D map resulting for the group source analysis are thresholded by the corresponding p-value<0.01. During the first 130 ms, not-consciously perceived peripheral fearful faces enhanced the neuronal activity in the right anterior medial temporal lobe, including parahippocampal gyrus and amygdala.

**Figure 2:**
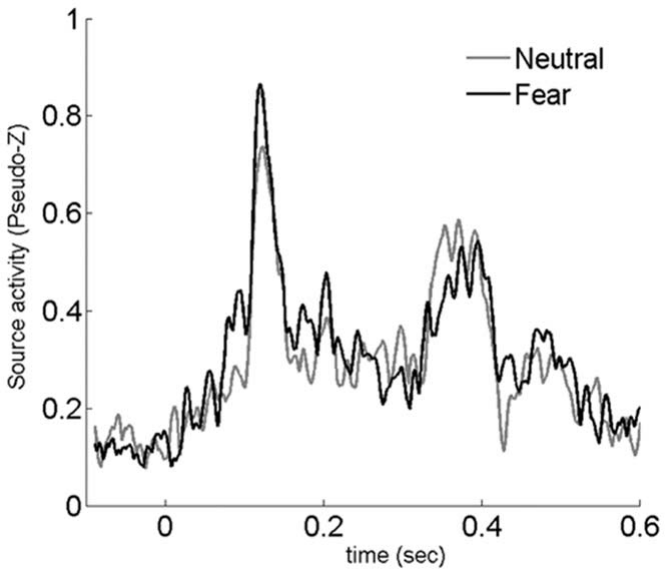
Time courses of activities at the right amygdala site. The activity elicited at the right amygdale site (21, −4, −15) by peripherally presented stimuli is depicted in black for fearful faces, in grey for neutral faces. The first peak appearing around 115 ms is stronger for fearful faces. The reported pseudo-Z values are taken from the subtraction of time course activities related to fearful faces minus those related to neutral faces in the corresponding voxels.

Table 2 lists the structures significantly more active for fearful than for neutral faces in the three latency windows when the unconsciously perceived faces were presented in the peripheral visual field. Only right-hemisphere regions were found in the first time window (80 to 130 ms): the right anterior temporal region including the uncus and the amygdala (Figure 1), the right pre- and post-central gyrus, the precuneus, the medial frontal gyrus and the anterior cingulate. To specify the time course of the right amygdala site, virtual sensors power source [33] were calculated individually and averaged. The resulting time courses of activation for neutral and fearful faces in the right amygdala (Talairach coordinates: 21, −4, −15) revealed a peak response in this structure around 115 ms (Figure 2). Source analyses showed that the activity around this peak was significantly larger for fearful faces. In the 140–190 ms time window, the right post and precentral gyrus and the right medial frontal gyrus remained significantly more activated for fearful faces (p<0.01), while the left inferior parietal and middle occipital regions became significantly more active for fearful faces (Table 2). In the 210–260 ms time window, two areas, left post-central gyrus and left supramarginal regions, were significantly more activated by fearful faces presented in the periphery (Table 2).

## Discussion

This study disclosed a quite early (before 130 ms) source activation difference in response to fearful versus neutral faces in the right anterior medial temporal region, including the amygdala, and in the anterior fronto-medial region, when presentation occurred in peripheral vision. When faces were presented centrally, regions along the ventral visual pathway (occipital cortex, fusiform gyrus, bilateral anterior temporal region) were more activated by fear than by neutral expression between 140 and 190 ms. Only after 210 ms, fronto-medial regions were also more activated by centrally presented fearful faces.

### Early right medio-temporal and medio-frontal cortex reaction to fear in periphery

Our results reinforce the hypothesis of an early (around 100 ms) modulation of brain activity by not consciously perceived facial expressions [34], and the involvement, in this processing, of a large distributed neural network, including amygdala and frontal regions [6], [18]. Most importantly, our data demonstrate for the first time that the advantage for fear expression occurs when not consciously perceived stimuli were presented peripherally. The network involved in rapid detection of danger is thus preferentially activated when danger appears in the periphery, stimulating mainly the magnocellular system. The magnocellular system role has rarely been studied in the emotional context, a unique study suggesting that different neural networks could be involved in emotional processing for central and peripheral vision [35]. However, it is demonstrated that low spatial frequency information carried by the magnocellular pathway is preferentially used for facial expression recognition [26], [27]. The present results provide further converging evidence that a rapid magnocellular pathway is implicated in fearful facial expression detection. The colliculo-thalamo-amygdalar pathway, tuned for visual information conveyed by magnocellular channels, has been proposed to carry fear-related stimuli [6], [23], and has already been reported to detect danger in animal studies [19]. The early amygdala response that we observed around 100 ms could result from this subcortical pathway activation for peripheral presentations. By stimulating the magnocellular system preferentially, the peripheral presentation of a related-to-danger stimulus would be more efficient than central presentation to activate a subcortical neuronal network able to quickly identify the potential danger coming from the periphery. The hypothesis that visual processing could be faster with the increase of eccentricity has already been formulated with simple visual stimuli [36], but to our knowledge, it is demonstrated here for the first time that emotional information can be processed faster in the peripheral visual field. This fast detection would result from magnocellular pathway recruitment, consistently with the subcortical pathway hypothesis.

The different brain regions sensitive to facial expressions in the first 130 ms are right-sided when the presentation is peripheral but are left-sided for central presentations. Hemispheric differences have been observed regarding emotional perception and particularly, a functional dissociation has been proposed between the right and left amygdala. The right amygdala would be more implicated than the left in a fast visual detection through a subcortical route [6]. The right-sided activity including amygdala's for detecting peripherally presented faces is an additional argument to point out a preference of the rapid subcortical visual pathway for danger coming from the periphery. The left-sided activity, between 80 and 130 ms for centrally presented faces, is limited to the inferior temporal region, confirming its role in a slower cortical visual analysis.

A possible limitation of our study could be the confidence of the MEG ability to detect sources as deep as the amygdala. Although some older papers have questioned the capacity of MEG to accurately detect and localize signals from deep neural structures [37]–[39], source reconstruction models are now routinely used to detect activity in deep structures including thalamic region [40], [41] and amygdala [42]–[47]. Thus, current whole-scalp sensor arrays are able to capture magnetic flux signals represented across the entire array that are also generated by deep sources [41]. The low MEG sensitivity to deep sources is a limit to study amygdala activations, but is also an argument that the observed sources correspond to strong activations, strong enough to be detected by MEG system despite its low sensitivity for this region. We cannot exclude an amygdala activity for central presentation, as reported in previous neuroimaging studies. But whether or not there is an amygdala activity for central presentation, this activity is too small to be detected by MEG, and so is smaller than the activity observed for peripheral presentation.

The frontal activation related to peripheral presentation of fearful faces before 130 ms could be driven by subcortical structures directly connected to the frontal lobe [48]. The frontal activation was located in the medial frontal gyrus but also encompassed the anterior part of the cingulate cortex (ACC). The ACC is involved in a wide range of cognitive functions including orientation of attention [49], [50], modulation and control of emotion [51]. Its implication has been demonstrated in the perception of task-irrelevant fearful faces [52] and in non-conscious facial expression perception [53], but its role remains debated. On the one hand, the ACC is activated only if the emotional information has to be ignored, which supports its role in the control of attention to affective stimuli [52]. On the other hand, its activation by very brief presentation of emotional faces is interpreted as its role in attention orientation [53]. Our results show ACC activation only for peripherally presented faces, when the emotional information was outside the usual attentional field. This supports the hypothesis that rostral ACC plays a role in directing attention toward the emotional information. The medial prefrontal cortex also participates in the regulation of emotional behaviours and autonomic response [54]. Early neuronal enhancement is reported in the frontal region in reaction to fearful faces [17], [55], [56] or other related-to-danger stimuli [57]. The ACC and medial prefrontal cortex activation only for peripheral fearful faces reinforces the hypothesis of their role in automatic response to danger [57] and is consistent with their function in shifts of spatial attention [58]. Emotionally salient stimuli would be more efficient than neutral stimuli to attract visual attention by recruiting the ACC and medial prefrontal cortex. Interestingly, the prefrontal cortex activity appears before 130 ms for peripherally presented faces but only after 210 ms for centrally presented faces, suggesting facilitation for attracting visual attention by peripheral apparition of danger. Thus the attentional shift may be triggered more rapidly and more efficiently by emotional stimuli occurring in the peripheral visual field better processed by a subcortical route and then by frontal structures.

### Visual ventral pathway reacts to fear in the center

Centrally presented fearful faces activated only the inferior temporal region in the first 130 ms of latency, and a larger neuronal network along the visual ventral pathway between 140 and 190 ms. During this time window, inferior occipital lobes, fusiform gyrus, superior temporal gyrus and inferior temporal lobe were sensitive to facial expressions only for central presentation. Those regions are part of a network known to be involved in face and facial expression processing [16]. Enhancement of activity for negative versus neutral expressions has been observed with different neuroimaging techniques in occipital lobes [59]–[62], fusiform gyrus [63]–[66], and superior temporal gyrus [67] particularly implicated in processing face changeable features like emotional expressions [16]. Even when they were subliminally presented, faces have been reported to activate this visual ventral network [34], [57], [68]. The present results confirm the sensibility of those regions to fearful faces, in particular faces presented in the central visual field. Contrarily to the fast and crude analysis performed by the subcortical route and the dorsal visual pathway, the ventral pathway proceeds to a detailed analysis of the stimulus.

This analysis conveyed by the parvocellular pathway is slower than the visual magnocellular system to reach the cortex [69], [70]. Thus a double functional dissociation is observed between the ventral visual cortical pathway which slowly and precisely processes centrally presented fearful faces and the subcortico-frontal route implicated in a crude and fast analysis of peripherally presented danger related stimuli.

### Other activities

During the 80–130 ms time window, a significant difference between fear and neutral face related activities was revealed in the right precuneus for peripherally presented faces only. This region is classically implicated in mental imagery [71], in visuo-spatial attentional shift [72], [73] and in processing emotional valence [74], [75]. The precuneus has abundant reciprocal connections with the anterior cingulate cortex, the dorso-lateral-prefrontal lobe and the temporal lobes [76], regions functionally linked to emotional valence rating [75].

The enhancement of activity in the precuneus for peripheral fearful faces can be interpreted as attentional resource recruitment. The important neuronal connection between precuneus and the ACC on the one hand, and their co-activation in the first stage of visual processing on the other hand, suggest that they belong to a same network, activated by emotional information in the peripheral visual field, allowing an early shift of attention to the stimulus and assessing its emotional valence.

The post-central gyrus showed stronger responses to fearful than to neutral faces in both spatial locations. However, for peripheral stimuli, the post-central gyrus is activated by fearful faces from 80 to 260 ms while for central presentation this activity appears only in the 140–190 ms time window. This somatosensorial region appears to play a role in emotion processing. Indeed, anatomical lesions or functional disturbance induced by transcranial magnetic stimulation of the right somatosensory cortex may be associated with impaired recognition of facial expressions, particularly fear [77], [78]. It remains debated whether the somatosensory cortex contribution to the emotional recognition is part of an early perceptual process. The somatosensory cortex has been found to be activated during explicit recognition of facial expression but not during gender judgment of expressive faces [79]. In our study, the somatosensory activity has been observed before 130 ms even though the emotional information was not consciously perceived. This result reinforces the hypothesis of an early perceptual role of the somatosensory cortex for emotional stimuli [80]. The early and sustained activation for peripherally presented faces suggests an implication of somatosensory areas not only in internal somatic representation of the emotion [77] but also in fast detection and reaction to danger.

Overall, the present data describe the spatiotemporal neuronal processing of fearful faces, not consciously perceived, presented in the peripheral visual field compared to central visual field. The fast reaction of the right medial temporal area is consistent with a role of the right amygdala in rapid and coarse detection of aversive stimuli coming from peripheral vision. This fast alert may convey subsequent frontal reaction crucial to shift attention towards peripheral threatening stimuli. This network preference for fear expression in the peripheral visual field may allow a more rapid behavioural response in dangerous situations, even without consciousness. An adaptive advantage is conferred by the fast automatic detection of potential threat outside the focus of attention, as danger in the external world mostly appears in the peripheral vision, requiring a rapid behavioural reaction before a conscious control.

## Materials and Methods

### Ethics statement

Each subject provided informed written consent. The study was conducted in accordance with the Declaration of Helsinki and was approved by the french ethics committee, Comité de protection des personnes SUD-EST IV, centre Leon Bérard.

### Subjects

Eleven healthy, right-handed subjects (6 women), aged 18 to 29 years (mean 22.9 yrs) participated in the study. None had a history of neurological or psychiatric disorders, and all had normal or corrected-to-normal vision. All provided informed written consent. The study was conducted in accordance with the Declaration of Helsinki and was approved by the local ethics committee.

### Stimuli and task

Each stimulus included three pictures, aligned horizontally, one picture in the center, one on the left and the right side, with 8° separating the central picture center from the peripheral picture center. The pictures were either faces or scrambled faces. We used eighty-four pictures of faces from the NimStim Face Stimulus Set [81] and ten scrambled faces. The selected faces consisted of 26 different individual faces (11 women), each with three different emotional expressions: neutral, happy and fear. The selected emotional faces had been categorized by emotion with more than 70% of accuracy, according to the NimStim validation table. In addition, five neutral faces, from other individuals, were used as masks (see protocol design below for details). The ten scrambled images were modified from the selected faces, scrambled with Adobe Photoshop software using a ripple distortion filter.

All pictures were black and white, resized and cropped to an oval shape. For face pictures, the oval crop was made inside the contour line of the face and all extra-facial information (hair around the face) was numerically erased. Eye position was controlled to ensure the same location within the oval across pictures. Mean luminance across pictures was equated. The final size of all the pictures was 5.6 to 7.5 cm subtending a visual angle of 4° to 5.2° at the viewing distance of 80 cm.

Each trial was beginning by a stimulus containing one face plus two scrambled faces. The stimuli were presented for 33 ms. In each stimulus, the face was presented centrally (50% of trials) or peripherally (50% of trials), half of them being a neutral face, the other half a fearful face Fifty percent of the faces presented in the periphery were in the right hemifield and 50% in the left one. The first stimulus was immediately followed by a mask, consisting of three neutral faces presented simultaneously and randomly chosen among the five neutral faces dedicated to the mask. The mask was presented for 200 ms and followed by a fixation cross presented in the center of the screen. There was a total of 624 trials, presented in 4 blocks using *Presentation* 6.0 ® software. The four conditions of 156 trials (neutral or fear expression, central or peripheral position) were randomly presented.

**Figure 3:**
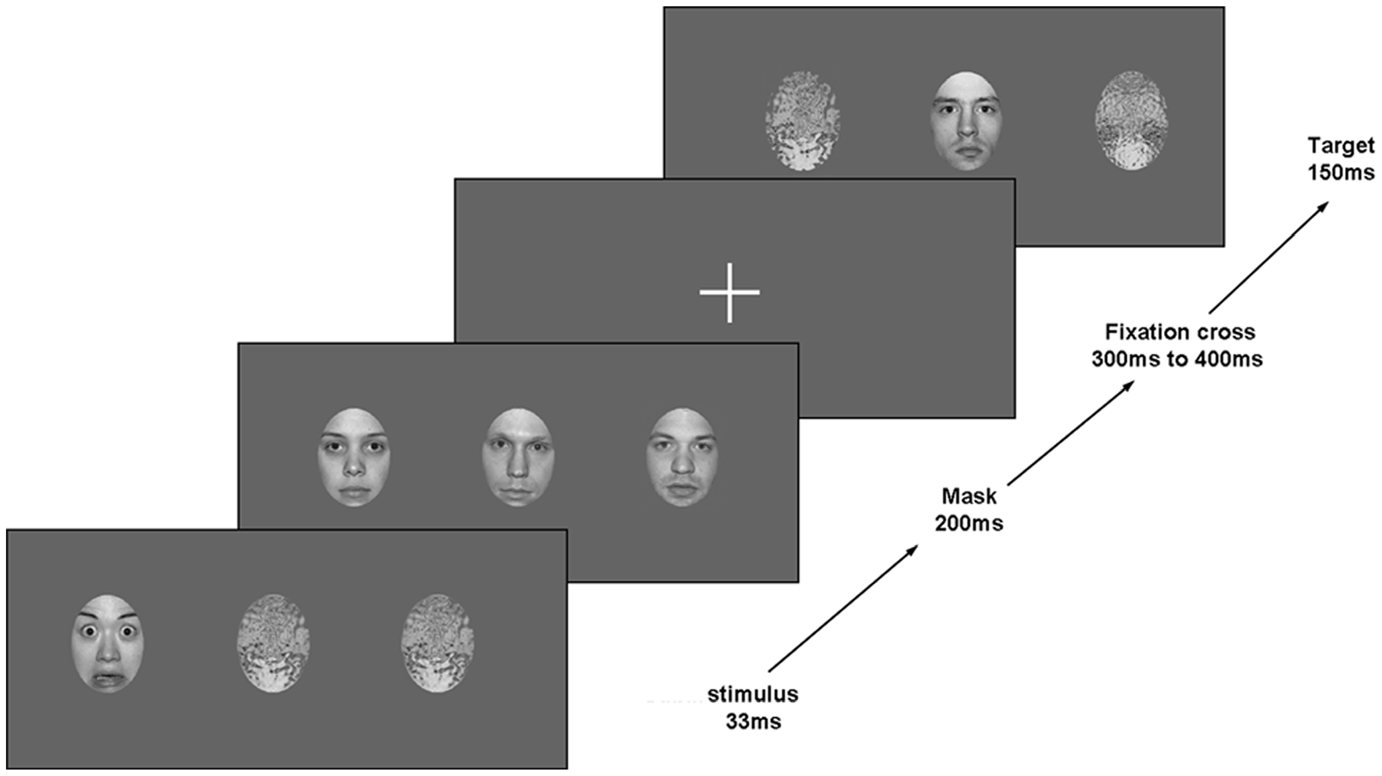
Example of trial. Each trial started with two scrambled faces and a fearful or neutral face presented for 33 ms, centrally or peripherally, immediately masked by 3 neutral faces. After a variable delay the target stimulus was appearing. The subject was asked to press a button when a happy face was occurring.

To ensure that subjects paid attention, a target stimulus was presented for 150 ms, 300 to 400 ms after the mask (Figure 3). The target stimuli contained one face and two scrambled images; 50% of the target faces were presented centrally and 50% peripherally. Three different expressions were presented as target stimuli: neutral, fear or happy, with equal probability. Subjects were asked to fixate the cross in the center of the screen and press a button when they detected a happy face in the target stimulus. Subjects were informed that each of the target stimuli were preceded by three faces (the mask), and were asked to focus their attention on the target stimuli to correctly perform the task. Only brain responses to the first stimulus (33 ms presentation) are presented in this paper.

The total inter-trial interval varied randomly between 1400 and 1600 ms. A fixation cross was present between the end of each target and the beginning of next trial. After the study, subjects were debriefed on what they had perceived. They all reported seeing only the 3 neutral faces (the masks) followed by the target stimulus. After we have informed them that in fact expressive faces were presented just before the three neutral faces, all subjects reported that they did not perceive this emotional stimulus, confirming that the analyzed stimuli, presented for 32 ms, were not consciously perceived by the subjects.

### MEG recording and co-registration with MRI

MEG signals were recorded on a CTF Omega 275 channel whole head system (VSM MedTech Ltd., Canada) in CERMEP, Lyon, France (www.cermep.fr). Continuous signals were recorded at 600 Hz using a third-order spatial gradient noise cancellation with an online bandpass of 0–300 Hz. Three fiducial coils (nasion, left and right pre-auricular points) were placed for each subject to determine the head position within the MEG helmet, and to provide co-registration with the anatomical MR images. Reference head position was recorded before the first block. Head position was controlled online during each block, and was readjusted to the reference position before each block, if necessary. The three coil positions were marked with radiology markers for the individual high resolution T1-weighted anatomical image (1.5 Tesla scanner, Siemens Sonata Maestro Class, 1 mm axial slices), ensuring an accurate MEG-MRI co-registration for source analyses.

During the task, eye movements were recorded by electrooculogram (EOG) to ensure that subjects were fixating the centre of the screen during the stimuli presentation. For all recorded subjects, the total number of eye blinking and horizontal eye movements during the 500 ms following the stimuli onset do not exceed 1% of the total number of presented trials.

### Source analyses

Event-related beamformer (ERB) source analyses [32], [33] were conducted on each subject's data for each of the four stimulus conditions (peripheral or central, neutral or fearful). In the present study, we used an adapted synthetic aperture magnetometry (SAM) beamformer algorithm [82]. As in other beamformer approaches, the SAM algorithm defines the signal of interest by the forward solution for a current dipole source at each target voxel. ERB uses the minimum-variance SAM beamforming algorithm on each trial and the forward solution for optimal current dipole direction to calculate a spatial filter for each voxel. The filters are noise-normalized, based on the spatial correlation present in the data. For each voxel, the resulting filter is then used to calculate the difference of source power between the baseline and the active window across time. We used the 100 ms pre-stimulus interval for the baseline. Finally, the resulting power source for the different analyzed time windows is expressed in a pseudo-Z value, defined as the difference of activity between the analyzed time window and the baseline, normalized by the noise. Contrary to the original SAM algorithm, the ERB method presently used allows analyses on narrow time windows, and is consequently adapted for early steps of cerebral processing analyses.

**Figure 4:**
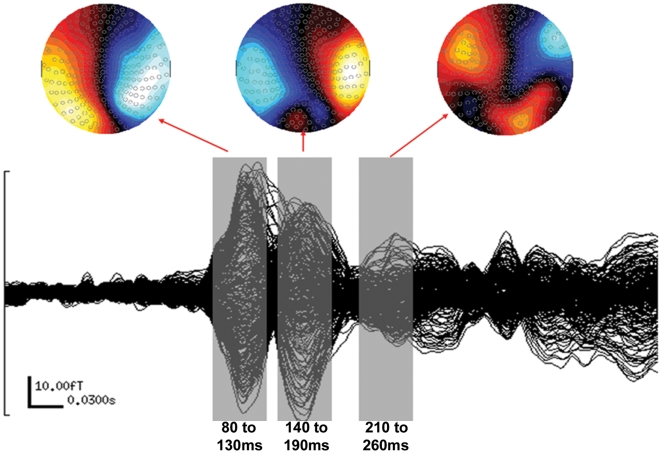
Sensor responses averaged across subjects and conditions. Event-related beamformer source analyses were performed in three 50 ms time windows (grey) surrounding the three major peaks. Magnetic activity maps represent the sensor activity for each maximum peak amplitude.

We applied the ERB analyses to three different time windows: 80–130 ms, 140–190 ms, and 210–260 ms. These time windows were chosen after a visual inspection of the average MEG signal. They corresponded to the three principal MEG components detected and the size of the windows (50 ms) encompassed the major part of these peaks (Figure 4).

For each condition, each subject and the three time windows, a 3-D power distribution map was calculated for the 1–30 Hz frequency band, using a 5 mm resolution reconstruction grid that encompassed the entire brain volume. The forward model for the beamformer calculation was based on a multi-sphere model fit to the inner skull surface extracted from the individual anatomical image with *Brainsuite2.0* software [83]. The 3-D ERB images were spatially normalized in an average brain (MNI 152), and put into Talairach stereotaxic space, using SPM2 (www.fil.ion.ucl.ac.uk/spm/software/spm2/), allowing statistical analysis and the computation of a group average of activation volumes. To take into account the brain anatomy variability and the individual brain normalization, group average activation maps were overlaid onto the average brain used for normalization.

Group analyses were completed with AFNI software [84]. A 2 by 2 within-subject ANOVA was performed on each analysed window to test the interaction effect between the spatial position factor (centre or periphery) and the facial expression factor (neutral or fearful faces). The resulting 3D map of the F values was used as a spatial mask for the comparison of the two facial expressions in the central or the peripheral presentation condition. For those 2 by 2 comparisons, only voxels with a significant interaction effect (uncorrected F<0.01) were analysed.

For each subject, each time window and for both stimulus spatial positions, contrast images between fearful and neutral faces were calculated voxel by voxel on the difference in power ratio between these two conditions. By subtracting the two conditions, uncorrelated noise was factored out of the resulting power ratio map. Resulting contrast images were tested by a one-sample t-test against zero. Differentially greater activation by fear relative to neutral condition was defined in the voxels with a significant factor interaction effect and a Student t-statistic exceeding an alpha level of 0.01.
